# A camera-phone based study reveals erratic eating pattern and disrupted daily eating-fasting cycle among adults in India

**DOI:** 10.1371/journal.pone.0172852

**Published:** 2017-03-06

**Authors:** Neelu Jain Gupta, Vinod Kumar, Satchidananda Panda

**Affiliations:** 1 Department of Zoology, University of Delhi, Delhi, India; 2 Department of Zoology, MMH College, Ghaziabad, Uttar Pradesh, India; 3 Salk Institute of Biological Sciences, La Jolla, California, United States of America; 4 UC San Diego Center for Circadian Biology, San Diego, California, United States of America; University of Alabama at Birmingham, UNITED STATES

## Abstract

The daily rhythm of feeding-fasting and meal-timing are emerging as important determinants of health. Circadian rhythm research in animal models and retrospective analyses of human nutrition data have shown that reduced length of overnight fasting or increased late night eating increases risk for metabolic diseases including obesity and diabetes. However, the daily rhythm in eating pattern in humans is rarely measured. Traditional methods to collect nutrition information through food diary and food log pay little attention to the timing of eating which may also change from day to day. We adopted a novel cell-phone based approach to longitudinally record all events of food and beverage intake in adults. In a feasibility study daily food-eating patterns of 93 healthy individuals were recorded for 21 days using camera phones. Analysis of the daily eating patterns of these individuals indicates deviation from conventional assumption that people eat three meals-a-day within a 12 h interval. We found that eating events are widespread throughout the day, with <30% of calories consumed before noon and >30% consumed in evening and late night hours. There was little difference in eating pattern between weekdays and weekends. In this cohort more than 50% of people spread their caloric intake events over 15 h or longer. One decile of the cohort who were spouses of shift-workers or had flexible work schedule spread their caloric intake over 20 h. Although the nutrition quality and diversity of food consumed is different between South-East Asian and Western countries, such overall disruption of daily eating-fasting rhythm is similar. Therefore, in view of hypothesis that disrupted daily eating pattern may contribute to the global increase in metabolic diseases and modification of daily eating pattern is a potential modifiable behavior to contain these diseases, monitoring eating pattern is an important aspect of lifestyle.

## Introduction

Animals and humans evolved on our rotating planet with predictable daily rhythms in activity-rest and associated rhythms in period of feeding and fasting. Accordingly, tissue-autonomous circadian rhythms have evolved to temporally coordinate activity-sleep and feeding-fasting to the appropriate time of the day-night cycle. This instructive pressure has resulted in daily oscillations in activities of thousands of genes in a tissue specific manner to offer metabolic fitness to the organism [1]. Industrialization and access to electrical lighting at night has changed human lifestyle from predominantly agrarian—with plenty of access to daylight and food during the day—to post-industrial lifestyle marked by predominantly indoor living and extended period of access to electrical lighting. This in turn has allowed humans to stay awake for longer periods of time under artificial light and have prolonged access to food.

There is a complex interaction between the quality of nutrition and circadian rhythms. In the most widely used animal model of obesity and chronic metabolic diseases, rodents are fed a high fat diet ad libitum (ad lib). This Diet-Induced Obesity (DIO) model also predisposes to diabetes, high cholesterol, fatty liver disease, and increased cardiovascular disease risks [2, 3]. These diseases are among the top ten causes of morbidity and mortality among adults in modern societies [4]. Interestingly, ad lib access to high fat diet also changes the daily eating pattern of rodents and they switch from a primarily nocturnal eating pattern to erratic eating habits throughout the day and night [5]. This erratic eating pattern under ad lib high fat feeding changes the global diurnal gene expression pattern in metabolic organs [6]. Conversely, time-restricted feeding (TRF) in which isogenic mice are given the isocaloric energy dense diet within an 8–12 h period are prevented from obesity, diabetes and associated metabolic diseases in both male and female rodents [7–10]. Even among diurnal animals, TRF prevents body weight gain and age- or diet- induced deterioration of heart function [11]. These animal studies in which detailed genomic and metabolic characterizations have elucidated the underlying molecular changes, raise a possibility for health improvement in human beings through temporal re-organization of daily food consumption.

Sleep, activity, and the associated eating pattern are overt outputs of circadian rhythms. Circadian and sleep disruption has long been associated with increased predisposition to obesity, diabetes and metabolic diseases [12–14]. Shift-work with erratic lifestyle also predisposes an individual to these diseases [15–17]. In humans, an aberrant eating pattern, such as late night caloric intake, increases the risk of developing coronary heart disease by as much as 55%, after controlling for diet and lifestyle [18]. These observations in humans are now replicated in controlled animal studies [19], lending further support to the notion that chronic circadian disruption contributes to risks for chronic diseases. Conversely, there is growing number of observations that constraining all caloric intake to <12 h can reduce breast cancer risk and improve prognosis [20, 21]. Similarly, in weight-loss studies, early ingestion of major meals during the day has been shown to enhance weight-loss [22]. These observations have prompted a renewed interest on daily pattern of food intake [23].

India is among the top 5 countries that account for more than 50% of the world’s diabetic patients [24]. Over the past 5 decades, it has rapidly moved from a largely agrarian society to an advanced economy with accelerated increase in electrification and increase in non-farm employment [25]. These changes in socio-economic parameters contribute significantly towards changes in circadian lifestyles and associated eating pattern. Contrary to conventional wisdom that humans in modern societies primarily eat three meals within a 12 h period, we hypothesize more than 50% of adults spread their caloric intake over >12 h. Specifically, an erratic eating pattern with food intake spread over a long period in the 24 hours within a day might exacerbate circadian disruption. If this behavior is sustained over years, it may increase the risk for metabolic diseases. Although 24-h food recollection, food diary and food frequency questionnaire are typically used in nutrition studies, they are not intended to capture daily eating pattern data and its day to day variation. Therefore, methods to collect evidence-based and time-stamped ingestion data and to analyze them for daily pattern of eating and fasting are being developed [26]. Camera phones are increasingly being used in nutritional studies [27, 28]. In this study, we tested the feasibility of monitoring daily eating pattern among healthy adults with no diagnosed disease using camera phones.

## Methods

This study was approved by the human ethics committee of MMH College, Ghaziabad, Uttar Pradesh. Participants were recruited through paper leaflets/flyers and notice board advertisements in public places. Inclusion criteria were as follows. Healthy adults 18 years or older, no- shift-work, no history of major sickness within the past 6 months, non-smoker, no diagnosed diabetes were included in this study. Female adults who were not pregnant or whose youngest child was older than 1 year, not enrolled in a weight-loss or weight-management program, not taking any medication that is meant for or has a known effect on appetite, no psychiatric disorder or on anti-depressant medicine, no out-of-town travel planned during the study period, no surgery in recent past were included in this study. Subjects were screened for inclusion and exclusion criteria by telephone and in-person interview. Based on profession (to screen out shift-workers) reported, subjects largely belonged to lower to medium income groups.

At the first visit, goals and methods of this study were described to the participants and those willing to participate in the program provided written consent. Each participant was given a functional camera phone and was asked to record all of his/her food, beverage and water intake using the camera function of the phone. In order to avoid potentially expensive data usage during transferring digital pictures, the subjects were asked to save the food pictures in their phone cameras. Subjects’ height and weight were measured using a calibrated scale and tape measure at the beginning and during their visit after the end of the 3 week monitoring period. At the end of the 3 week period, a follow-up questionnaire was filled by each participant. Participants were nominally compensated for their time and effort in this study.

In order to monitor eating pattern in this study, data were collected from a Tuesday/Wednesday midnight of the first week to Tuesday/Wednesday midnight three weeks later. Subjects received instructions to record every item consumed (food, drink, water), regardless of serving size, using the camera phone. Participants received a phone call or text message at random times during the day, reminding them to log their food data. The leftovers from items that were not completely consumed were to be recorded again and noted. Participants that fell sick and required regular medications or diet modifications were removed from the study.

After 21 days, the pictures from their camera were downloaded and analyzed for time stamp and food pictures. All participants were re-contacted to verify one or more of three specific questions about the data; any unrecognizable food picture, whether they observed any religious fasting on days with <3 records, and abnormal eating pattern resembling that of a shift-worker. As many individuals in India follow religious practices to observe partial-, complete- fast, or modified diet on religious days or festivities, days with less than 3 total events (including non-caloric content items) were flagged and verified with the participants for any observed fasting day or whether they forgot to log data. If they had <3 events and the reason was failure to record data, those days were removed from analyses. Individuals with erratic eating pattern (most of the food items consumed very late at night, or a switch from day to night time eating on 3 or more days of the week) were asked if they or someone in their family does shiftwork or flexible work that would explain such eating pattern. Each participant was assigned a random alpha-numeric code and the food picture names were tagged with the participant ID as a prefix. Each picture was renamed using windows ImageRenaming application to include anonymous name and time-stamp in the format: SubjectID_YYYY-MM-DD_HH-MM-SS. Each picture was sorted into water or non-water items. The name and portion size of food items were independently assessed by three researchers. A fourth researcher verified any discrepancy between annotations or randomly checked the images and the annotations. The non-water items were further annotated by the research team by looking up Food And Nutrient Database For Dietary Studies (FNDDS) website of USDA National Nutrient Database for Standard Reference [27]., CalorieKing and MyFitnessPal websites. The FNDDS has caloric values for several food items commonly consumed in India (fruits, raw vegetables, rice, bread, coffee, tea, milk etc.). Some of the foods consumed in India may have different names, but they have similar composition to several FNDDS listed items (e.g. channa in India is garbanzo beans, Daal is lentil soup, Bhel is rice crispie etc.). When a food item or its equivalent was not found in FNDDS, it was looked up in CalorieKing or MyFitnessPal websites (e.g. Dosa, samosa, vada).

All data were imported to GraphPad Prism for subsequent analyses and statistical tests. Time of caloric events for each participant was analyzed in GraphPad Prism to derive 95 percentile eating interval or “eating duration”. Time of caloric events for each individual was also plotted as a scatter plot to visualize the spread of caloric events within 24 hours. Beginning of the eating duration was considered as breakfast or time of first caloric intake and the end of the duration was considered the last caloric intake. Time of eating at all events from all participants was pooled and frequency distribution of eating events in one hour bin over 24 h of the day was derived. For each individual, difference between two consecutive events was calculated and if it was less than15min, it was considered as one meal. Frequency distribution of all inter-meal intervals was calculated to find the 25-, 50-, and 75 percentile intervals.

Resting energy expenditure for each individual was calculated using modified Harris Benedict equation [29]. Reported daily caloric intake for each participant was calculated from their food pictures.

## Results

**Fig 1:**
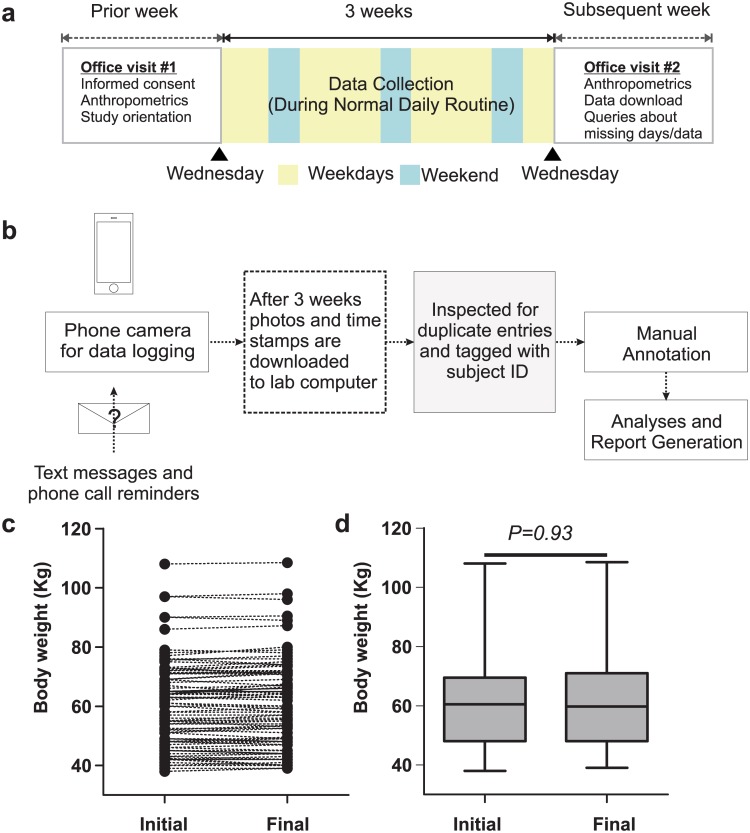
A simple camera phone based method to collect human nutrition information. (A) Schematic of the study to collect eating pattern data for 3 weeks. (B) Work flow for data collection, download and annotation. Reminders were sent to the user’s preferred phones at random time of the day. Although the user could potentially review collected data, it did not affect their body weight at baseline and after 3 weeks of data collection as seen in individual body weights (C) and (D) in median body weight (±max/min range, paired t-test p>0.05).

In India, the vast diversity of non-standard food items, language diversity, and insufficient awareness of portion size, makes it difficult to adopt standard method of nutrition studies in which the participants are asked to describe food and portion size. Therefore, we adopted a smartphone-based method to collect images of food that transfers the burden of food annotation from users to the researchers (Fig 1A and 1B). We used the picture taking function of smartphones to collect food consumption data from a cohort of healthy males and females in National Capital Territory of India (Delhi-NCR)—a cosmopolitan city of India. Out of 102 participants who consented to the study and started the data collection, eight dropped out due to study-unrelated sickness, leaving 94 participants who completed the study (Table 1). There was no significant reduction in the body weight of participants during the 3 weeks self-logging of nutrition data (Fig 1C and 1D), suggesting that self-reporting did not adversely affect food intake leading to significant weight loss.

**Table 1:** Composition of the cohort, their anthropometric measurements before and after the observation period, and estimated energy intake during observation period.

	Male	Female	All
n	35	59	94
Age	36.97	31.51	33.54
	(19–58)	(19–52)	(19–58)
Height	169.81	157.45	162.05
	(149.86–192)	(124.36–176)	(124.36–192)
Initial BMI (before baseline)	23.9	22.19	22.83
	(16.19–32.02)	(14.84–34.78)	(14.84–34.78)
Final BMI (after baseline)	24.07	22.16	22.87
	(15.55–31.9)	(15.23–34.42)	(15.23–34.42)
Change in BMI (after baseline)	-0.1724	0.02615	-0.0478
Paired t-test P-value	p = .036; “t = 2.175 df = 34”	ns	ns
Calculated REE (Kcal)	1623	1321	1422
	(1378–1467)	(1294–1347)	(1540–1706)
Average daily caloric intake reported (Kcal)	1347	1320	1329
	(1230–1464)	(1247–1393)	(1268–1390)
Percentage of REE intake reported	83.86	100.2	94.71
	(76.19–91.52)	(94.52–106.0)	(89.91–99.51)

Average and 95% confidence interval values (in parentheses) are shown. Paired t-test p values for body weight before and after observation period were >0.05 (not significant). Resting energy expenditure was calculated for each individual using the modified Harris Benedict equation [29].

**Fig 2:**
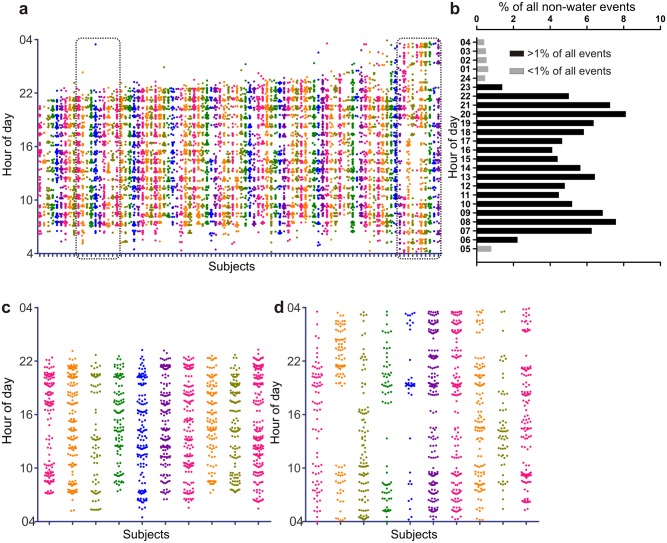
Daily eating pattern is highly erratic at individual level. (A) Scatter plot of all non-water events collected from 94 healthy subjects, where each vertical array is color coded for time-stamped ingestion events from each subject over 21 days. The subjects are arranged from left to right with increasing portion of 24 h day where they ingested food or beverages. (B) Frequency distribution of all non-water ingestion events as percentage of total number of events binned in hourly interval shows very low intake at cohort level during midnight-5am and three peaks approximately corresponding to the conventional breakfast, lunch and dinner. However, at individual level, the day to day variations in eating pattern blurs these pattern both among (C) subjects who eat during a shorter time interval or those (D) who spread their ingestion events throughout a 24 h period. Subjects in C and D are subsets of data shown in (A). Each column in Fig 2a,c,d represents data from individual subject.

The use of camera phone along with daily reminders to record ingestion events was effective as we could collect 1940 days of eating data out of (94 participants*21 days) 1974 days of intended data collection. Out of 17622 total images collected from 94 participants over a 3 week period, 7473 pictures contained only water, 5990 contained only one food/beverage and the rest contained multiple food and/or beverages. Unlike in the Western countries, low calorie soda is not marketed nor consumed to a great extent in India. The most common beverages consumed in India are tea or coffee with milk and sugar. So, all non-water consumptions were considered caloric containing beverages. The time-stamps of non-water items, numbering 17238, were used to illustrate the eating pattern of all participants. This translates to ~8.5 ingestion events/day, which is not unusual as people typically drink tea/coffee/beverages and snacks several times a day. Time-stamps of all non-water ingestion events over 21 days were plotted in a scatter plot for each individual along the vertical axis (Fig 2A). If subjects ate three meals a day and were consistent with their meal times, one would expect to observe the time-stamps cluster into three clusters. However, if the subjects ate more than 3 calorie-containing meals a day or if the timing of three meals was random on different days, the time-stamps would scatter throughout the day. In contrast to the self-reported 3 meals/day structure of meals from most of the participants, a breakfast-lunch-dinner temporal pattern was largely absent (Fig 2A, 2C and 2D).

The non-water events were binned into 1 h interval over 24 h and were found to populate a large segment of the 24 h day (Fig 2B) leaving only 6 h between 11 pm and 5 am when the number of events/h were <1% of total events (Fig 2B). As has been found in a comparable study in the US [26], we considered 4 am as the onset of “metabolic day”, so that consumption from 4 am to 3:59:59 am was considered a 24 h day. Although at individual participant level three clusters of food intake was largely absent, when all ingestion events from the entire cohort was analyzed together, there were three peaks of events at 8 am, 1 pm, and 8 pm (Fig 2B). Each of these three peaks accounted for ≤8% of all events and the fraction of all events in every hour was >4% in a contiguous period of 16 h spanning 7 am-10 pm (Fig 2B). During this 16 h interval ingestion events also accounted for >5%/h except at 3 pm and 4 pm. A parsimonious interpretation of such temporally widespread pattern of caloric intake is that the individuals ate more frequently and/or had large day to day variation in their ingestion events. Upon arranging the eating pattern of all participants according to increasing duration of the day throughout which they spread their caloric intake, the top decile were found to spread their caloric intake throughout the 24 h (Fig 2D). Three subjects (marked as * in Fig 2D) logged <5% of all their non-water ingestion events between 10 am-6 pm and in follow-up interview with these participants, they confirmed an erratic lifestyle of being stay-at-home spouses of shift-workers, even though they themselves were not employed as shift workers.

**Fig 3:**
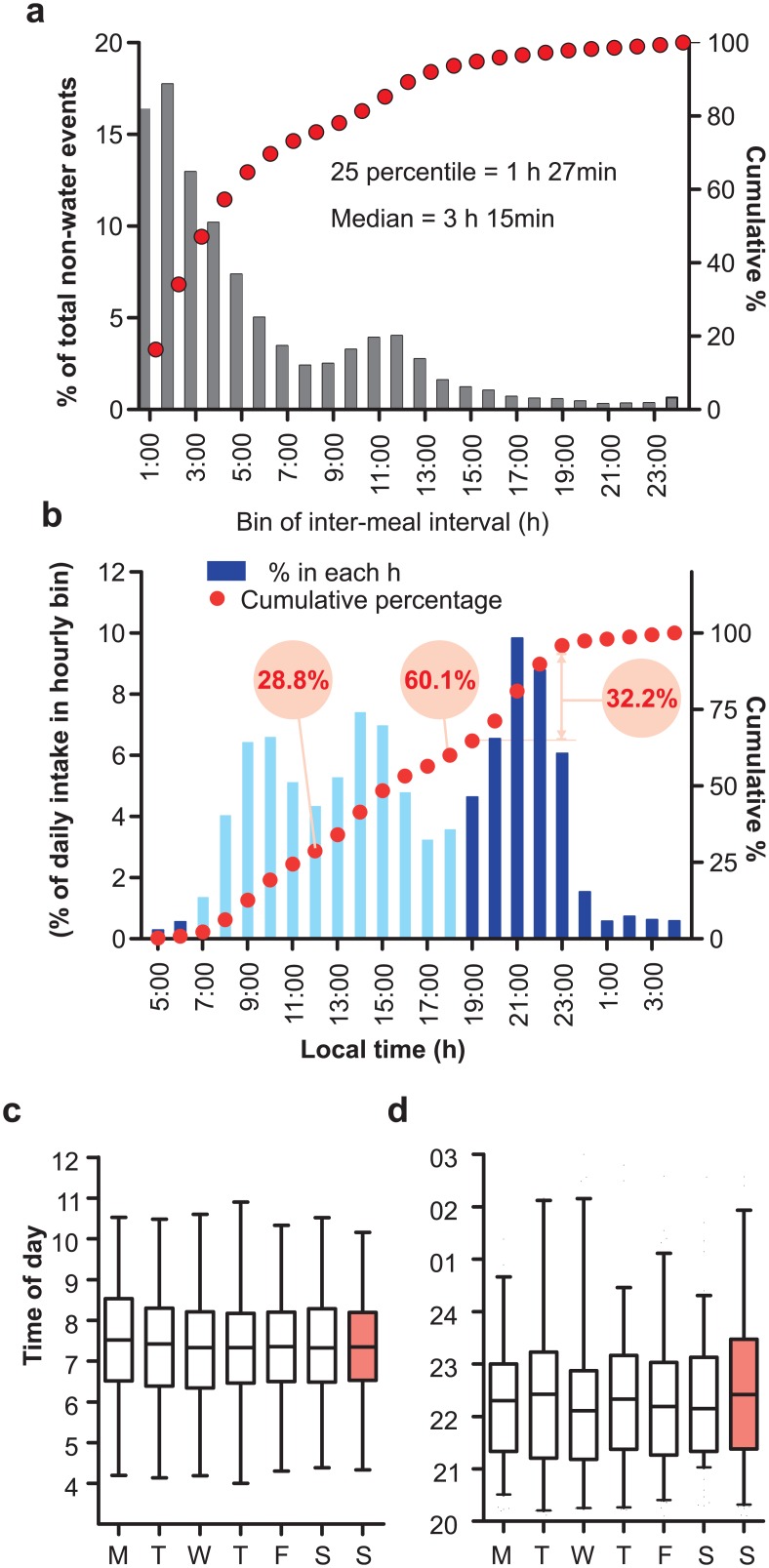
Temporal aspects of daily eating pattern. (A) Frequency distribution of time interval between two consecutive non-water ingestion events. Events with <15min intermeal intervals were considered as one meal and were not included in this analysis. Extended intermeal intervals of >16 h constituted a small fraction of data points and were likely due to cultural fasting or the subjects forgetting to log their food. (B) Percentage of all caloric intake in hourly bins show three prominent peaks corresponding to conventional times for breakfast, lunch and dinner. However, subjects consumed more food at dinner than at breakfast or lunch. (C) The day to day variation in breakfast or dinner time for the cohort (median+25%ile and min-max range) showed no significant difference between days. However, there was larger variation for dinner than for breakfast time.

As has been shown earlier [26], we considered all non-water events recorded within 15 min of each other as part of one meal. At group level, 25% of all meals were within 1 h 27 min of another meal and the median inter-meal interval was 3 h 15 min. Only 25% of the meals occurred after > 7 h 44 min of fasting (Fig 3A). The post-prandial physiological response marked with an elevation in blood glucose and insulin action for absorption and nutrient utilization in anabolic metabolism can last for >90min. So, when ingestion events occur within 90 min of a previous meal, physiology likely sustains at an anabolic state between meals.

As every food image was annotated for estimated caloric content, we tested the temporal pattern of caloric intake by this cohort. The fraction of total calories consumed in every hourly bin starting after 4 am showed three clear peaks that followed the temporal pattern of a number of reported events (Figs 2B and 3B). Given that we considered 4 am as the start of a metabolic day, in the first 8 h or by noon, the cohort consumed only 28.8% or less than 1/3^rd^ of the daily caloric intake. By 6 pm, they consumed 60.1% and in the 4 h of evening between 7 pm-11 pm, they consumed more calories (32.2%) than in the first 8 h of the day (Fig 3B). In summary, there was a clear trend towards larger portion of daily caloric intake being consumed at night.

**Fig 4:**
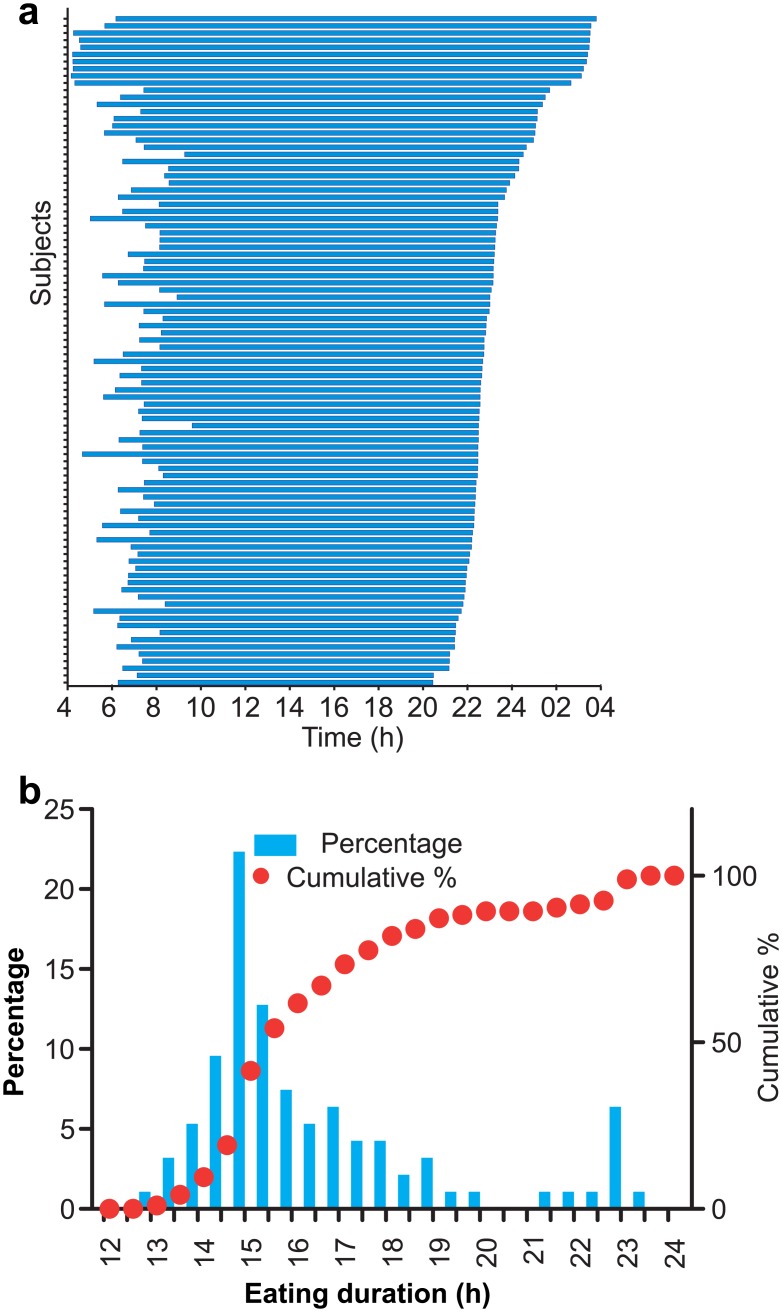
Eating duration for majority of individuals is longer than 12h. (A) Eating duration of 94 participants arranged with increasing time of last meal. (B) Frequency distribution of eating duration in 30min bins and cumulative percentage shows ~60% of the study cohort eat for 15 h or longer.

**Fig 5:**
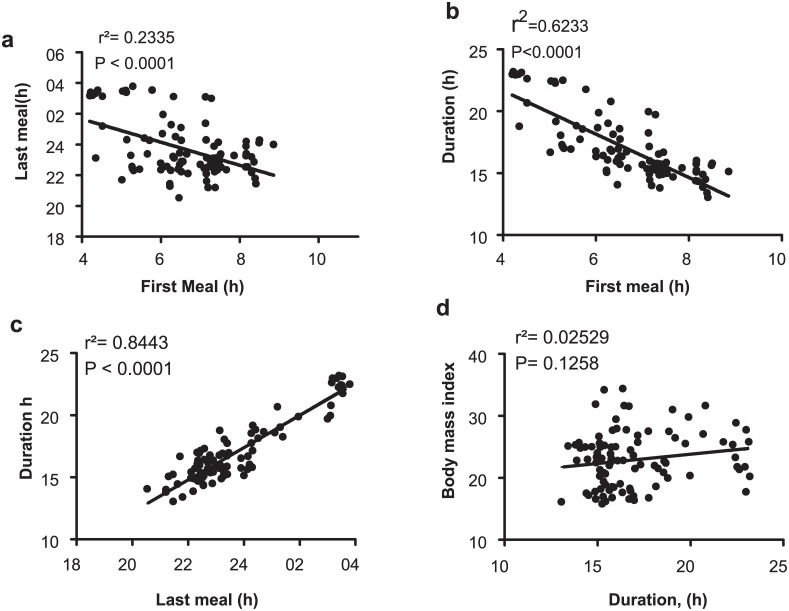
Inter-relation of various daily eating parameters. The timing of first meal inversely correlates with (A) the timing of last meal and with (B) duration. (C) The time of last meal strongly correlates with the eating duration. However, the eating duration does not show a simple correlation with BMI.

Having observed the surprisingly large variance in the first and last caloric intake and the absence of a clear 3 meals/day eating pattern at individual level (Fig 2A, 2C and 2D), a better description of eating pattern would be the daily interval when a person is likely to eat. So we defined the eating duration as the time interval (4 am onwards) that contained 95% (2.5%ile-97.5%ile) of all intake events during the monitoring period (Fig 4A). The median eating duration was 15.53 h with 25-75th percentile interval being 15.05–17.42 h. The top decile with erratic eating pattern during the monitoring period (Fig 2D) had an eating duration of >20h (Fig 4B). Beginning of the eating interval approximates the likely first caloric intake and the end of the duration corresponds to the last caloric intake of the day. Overall the median breakfast and time of last caloric intake were 6:58 am (6:10 am-7:27 am; 25-75th percentile interval), and 10:45 pm (10:18 pm– 11:58 pm; 25-75th percentile interval). Unlike what was observed in a comparable US study [26], there was no statistically significant difference in the median breakfast or dinner time between weekdays and weekends. The time of first caloric intake negatively correlated with the last caloric intake (r^2^ = 0.2335, p<0.0001) (Fig 5A). Eating duration positively correlated with the time of last caloric intake (r^2^ = 0.8443) (Fig 5B), while it inversely correlated with the time of breakfast (r^2^ = 0.6233) (Fig 5C) or with BMI (r^2^ = 0.017) (Fig 5D). The weak correlation (r^2^ = 0.025, P = 0.1258) between the eating duration and BMI could be due to the smaller sample size, heterogeneity of participants in terms of gender and age, and the fact that the eating pattern recorded in the monitoring period is a short-term snapshot of a person’s diet-related behaviors. In summary, in contrast to the conventional thinking that humans eat 3 meals a day within approximately 12 h interval, the study found both intra-individual and inter-individual variations in daily eating pattern.

## Discussion

We report a feasibility study to use camera phones as a method to collect evidence-backed nutrition metadata containing what, when, and how much a person eats in a developing country. Such studies have been done in controlled settings with users meticulously placing a size/color reference next to the meal, taking a picture from a recommended angle and distance [28]. However, in free living conditions, such methods may not be feasible for longitudinal studies where daily eating pattern and day to day or weekday-weekend variability is a parameter to be assessed. While smartphones have been ubiquitous with fast internet connectivity and relatively cheap data plans, in many developing countries like India, the use of fully functional smartphones as nutrition data collection devices has yet to be established. Therefore, we tested whether relatively cheap camera phone can be used as an alternate device to collect nutrition metadata. We found that in India the participants were as meticulous in recording data as participants in the US. The number of data points collected per individual in India is equivalent to what was observed in a recent study using smartphones in the US [26]. Nevertheless, it is difficult to estimate the false negative (when an individual ingests and forgets to record) reporting and we cannot rule out the possibility that some fraction of actual intake was not reported. In a recent study using smartphones, a random push notification and user’s response to the notification was used to estimate false negative reporting to be ~10% [26]. It is reasonable to assume that the actual energy intake for moderately active individuals would be higher than the resting energy expenditure (REE). The reported daily caloric intake was only 83.86% of REE for men and 100.2% of REE for women (Table 1), which indicates some caloric intake was not reported.

The data collection method appears to have no immediate impact on reducing energy intake that would result in weight loss. Unlike in the US study, where the participants could not review their food data during the data collection period, the participants in this study could easily review their own nutrition data stored in the local phone. But they were advised not to review the food pictures stored in their phones. We found that there was no acute effect of this potential user nutrition data review on reducing food intake, as there was no significant decline in body weight in this cohort (Fig 1C and 1D). Therefore, this method has potential for adoption in developing and under-developed countries as a data collection method. This is of significant importance as more than half of world’s metabolic disease patients live in these countries and nearly 78 million diabetic patients live in India [24]. Furthermore, frequent malnutrition in some of these countries is also a risk for stunted growth and increased disease susceptibility. As both nutrition and eating pattern are increasingly recognized as modifiable factors for alleviating these disease risks, methods to collect nutrition data in these countries is urgently needed.

Furthermore, people in India and in many developing countries eat a diverse range of home-cooked food with very little similarity to the standardized recipes or ready to eat pre-packaged food items consumed in the Western countries. Therefore, it is often difficult to find nutrition values for food consumed in developing countries. By adopting an image-based data collection system, we could overcome the barrier to food data collection which typically requires matching food data to a standard library and assessing portion size. We acknowledge the lack of accurate nutrition values for some items may introduce inaccuracy, but it is also a powerful survey tool to collect the diversity of food items consumed, so that effort can be focused on characterizing the most frequently eaten food.

We found many similarities and differences in the eating pattern of comparable sized non-shiftwork cohorts (of equivalent age and gender composition) in the US and in India. In both countries we found people don’t restrict their daily nutrition intake to breakfast, lunch and dinner. Rather, in addition to these three meals, there is frequent caloric intake throughout the 24 h day. In the US cohort, parallel measurement of sleep by actigraphy devices showed that reduced sleep correlated with frequent eating throughout the wakeful hours. Although we did not objectively measure sleep, we also found erratic eating pattern covered a large portion of the 24 h day. In the US cohort >50% adults spread their caloric intake over 15 h or longer, and in the present study a similar trend was found. Surprisingly, although one of our exclusion criteria was shift-work, we found nearly 10% of the cohorts had an extremely erratic lifestyle similar to that of shift-work; their eating time was spread well over 20 h during the 3 weeks observation period. Upon close examination we found they included spouses of shift-workers and people employed with flexible hours. This is of specific health interest as these two subgroups are generally not included in national surveys on shift-work. While shift-work is known to be a risk factor for several non-infectious chronic diseases including cancer, the extent of circadian disruptions among flexible workers and of family members of shift or flexible workers and their disease risks is rarely addressed. As the developing countries like India have a sizeable portion of shift-workers and flexible hour workers, erratic lifestyle in these unaccounted subgroups can be a hidden population risk for NCDs.

**Fig 6:**
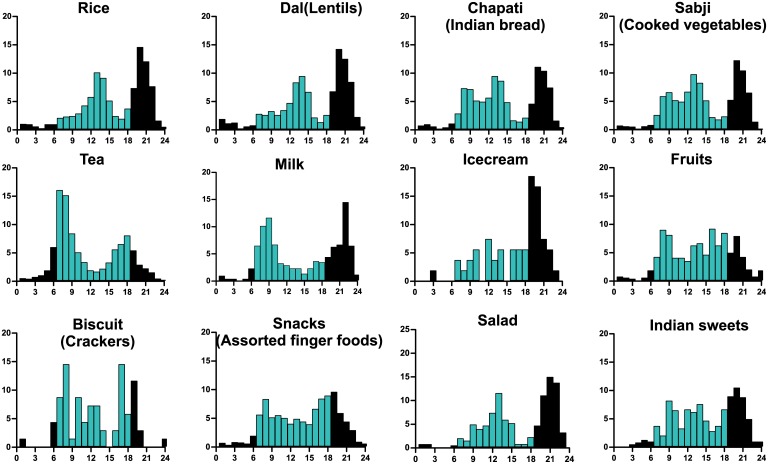
Daily consumption pattern of different food shows a unique pattern. Percentage of pictures containing a given food item in hourly bins are shown. Data for the presumptive daytime from 6am to 6pm is shown in cyan blue.

We also found a nocturnal shift in eating pattern both in the US and in India. While among the US cohort, <25% calories were consumed by noon and people consumed more than 1/3^rd^ of their daily caloric intake after 6 pm, in our study people consumed ~28% of their calories by noon and ~40% food after 6 pm. Specifically, between 7–11 pm, the cohort ate more than 30% of daily caloric intake (Fig 3B). Importantly, the diversity of food consumed during day or night was distinct; high glycemic foods such as cooked rice, ice cream, Indian sweets were consumed at night (Fig 6). This nocturnal shift of caloric intake combined with the recent finding that melatonin can suppress post-prandial insulin release [30], raises the hypothesis that the nocturnal food intake might contribute to rising diabetes trend in both countries [24]. There was also a remarkable difference in weekday and weekend eating pattern in two populations. While in the US, the cultural normal is at least 2 days of weekend off days, in India 6 working days is more of a norm in non-farm employment. Accordingly, in the Indian cohort we did not see any significant shift in breakfast time during the weekend as was reported in the US cohort. They showed a slight delay in time of dinner consumption on Sunday, which was not statistically significant (Fig 3C).

Despite the observed prevalence of erratic eating pattern in both countries, there was no simple correlation between eating duration and BMI in either study. Although increased BMI is considered a risk for metabolic diseases, it does not always correlate with diabetes [31] or cardiovascular diseases [32]. Even rodents that are fed a high fructose or high sucrose diet, may eat erratically and develop type2 diabetes without gaining excessive body weight. Glucose intolerance and metabolic diseases in these normal weight mice is prevented by a short daily eating duration [8]. Similarly, a review of time-restricted feeding in both rodents and humans has shown metabolic health benefits may arise from TRF in controlled studies without significant reduction in body weight [33]. Therefore, future studies with larger cohorts and broader evaluation of disease states is necessary to assess the contribution of eating pattern along with diet type to the risk for metabolic diseases.

## References

[1] Zarrinpar A, Chaix A, Panda S. Daily Eating Patterns and Their Impact on Health and Disease. Trends Endocrinol Metab. 2016;27(2):69–83. Epub 2015/12/27. 10.1016/j.tem.2015.11.007 26706567 PMC5081399

[2] Unger RH. Lipotoxic diseases. Annual review of medicine. 2002;53:319–36. Epub 2002/01/31. 10.1146/annurev.med.53.082901.104057 11818477

[3] Lin S, Thomas TC, Storlien LH, Huang XF. Development of high fat diet-induced obesity and leptin resistance in C57Bl/6J mice. Int J Obes Relat Metab Disord. 2000;24(5):639–46. Epub 2000/06/13. 10849588 10.1038/sj.ijo.0801209

[4] Lozano R, Naghavi M, Foreman K, Lim S, Shibuya K, Aboyans V, et al Global and regional mortality from 235 causes of death for 20 age groups in 1990 and 2010: a systematic analysis for the Global Burden of Disease Study 2010. Lancet. 2012;380(9859):2095–128. Epub 2012/12/19. 10.1016/S0140-6736(12)61728-0 23245604 PMC10790329

[5] Kohsaka A, Laposky AD, Ramsey KM, Estrada C, Joshu C, Kobayashi Y, et al High-fat diet disrupts behavioral and molecular circadian rhythms in mice. Cell metabolism. 2007;6(5):414–21. Epub 2007/11/07. 10.1016/j.cmet.2007.09.006 17983587

[6] Eckel-Mahan KL, Patel VR, de Mateo S, Orozco-Solis R, Ceglia NJ, Sahar S, et al Reprogramming of the circadian clock by nutritional challenge. Cell. 2013;155(7):1464–78. Epub 2013/12/24. 10.1016/j.cell.2013.11.034 24360271 PMC4573395

[7] Hatori M, Vollmers C, Zarrinpar A, Ditacchio L, Bushong EA, Gill S, et al Time-Restricted Feeding without Reducing Caloric Intake Prevents Metabolic Diseases in Mice Fed a High-Fat Diet. Cell metabolism. 2012;15(6):848–60. Epub 2012/05/23. 10.1016/j.cmet.2012.04.019 22608008 PMC3491655

[8] Chaix A, Zarrinpar A, Miu P, Panda S. Time-Restricted Feeding Is a Preventative and Therapeutic Intervention against Diverse Nutritional Challenges. Cell metabolism. 2014;20(6):991–1005. Epub 2014/12/04. 10.1016/j.cmet.2014.11.001 25470547 PMC4255155

[9] Sherman H, Genzer Y, Cohen R, Chapnik N, Madar Z, Froy O. Timed high-fat diet resets circadian metabolism and prevents obesity. FASEB J. 2012;26(8):3493–502. Epub 2012/05/18. 10.1096/fj.12-208868 22593546

[10] Chung H, Chou W, Sears DD, Patterson RE, Webster NJ, Ellies LG. Time-restricted feeding improves insulin resistance and hepatic steatosis in a mouse model of postmenopausal obesity. Metabolism. 2016;65(12):1743–54. Epub 2016/11/12. 10.1016/j.metabol.2016.09.006 27832862 PMC5123758

[11] Gill S, Le HD, Melkani GC, Panda S. Time-restricted feeding attenuates age-related cardiac decline in Drosophila. Science. 2015;347(6227):1265–9. Epub 2015/03/15. 10.1126/science.1256682 25766238 PMC4578815

[12] Spiegel K, Leproult R, Van Cauter E. Impact of sleep debt on metabolic and endocrine function. Lancet. 1999;354(9188):1435–9. Epub 1999/10/30. 10.1016/S0140-6736(99)01376-8 10543671

[13] Knutson KL, Spiegel K, Penev P, Van Cauter E. The metabolic consequences of sleep deprivation. Sleep Med Rev. 2007;11(3):163–78. Epub 2007/04/20. 10.1016/j.smrv.2007.01.002 17442599 PMC1991337

[14] Arble DM, Bass J, Behn CD, Butler MP, Challet E, Czeisler C, et al Impact of Sleep and Circadian Disruption on Energy Balance and Diabetes: A Summary of Workshop Discussions. Sleep. 2015. Epub 2015/11/14. 10.5665/sleep.5226 PMC4667373 26564131

[15] Sookoian S, Gemma C, Fernandez Gianotti T, Burgueno A, Alvarez A, Gonzalez C, et al Effects of rotating shift work on biomarkers of metabolic syndrome and inflammation. Journal of internal medicine. 2007;261(3):285–92. 10.1111/j.1365-2796.2007.01766.x 17305651

[16] Brown DL, Feskanich D, Sanchez BN, Rexrode KM, Schernhammer ES, Lisabeth LD. Rotating night shift work and the risk of ischemic stroke. Am J Epidemiol. 2009;169(11):1370–7. Epub 2009/04/10. 10.1093/aje/kwp056 19357324 PMC2727250

[17] Davis S, Mirick DK, Stevens RG. Night shift work, light at night, and risk of breast cancer. Journal of the National Cancer Institute. 2001;93(20):1557–62. Epub 2001/10/18. 11604479 10.1093/jnci/93.20.1557

[18] Cahill LE, Chiuve SE, Mekary RA, Jensen MK, Flint AJ, Hu FB, et al Prospective study of breakfast eating and incident coronary heart disease in a cohort of male US health professionals. Circulation. 2013;128(4):337–43. Epub 2013/07/24. 10.1161/CIRCULATIONAHA.113.001474 23877060 PMC3797523

[19] Lucassen EA, Coomans CP, van Putten M, de Kreij SR, van Genugten JH, Sutorius RP, et al Environmental 24-hr Cycles Are Essential for Health. Curr Biol. 2016;26(14):1843–53. Epub 2016/07/19. 10.1016/j.cub.2016.05.038 27426518

[20] Marinac CR, Nelson SH, Breen CI, Hartman SJ, Natarajan L, Pierce JP, et al Prolonged Nightly Fasting and Breast Cancer Prognosis. JAMA oncology. 2016. Epub 2016/04/01. 10.1001/jamaoncol.2016.0164 PMC4982776 27032109

[21] Marinac CR, Natarajan L, Sears DD, Gallo LC, Hartman SJ, Arredondo E, et al Prolonged Nightly Fasting and Breast Cancer Risk: Findings from NHANES (2009–2010). Cancer epidemiology, biomarkers & prevention: a publication of the American Association for Cancer Research, cosponsored by the American Society of Preventive Oncology. 2015;24(5):783–9. Epub 2015/04/22. 10.1158/1055-9965.EPI-14-1292 PMC4417458 25896523

[22] Garaulet M, Gomez-Abellan P, Alburquerque-Bejar JJ, Lee YC, Ordovas JM, Scheer FA. Timing of food intake predicts weight loss effectiveness. Int J Obes (Lond). 2013. Epub 2013/01/30. 10.1038/ijo.2012.229 PMC3756673 23357955

[23] Longo VD, Panda S. Fasting, Circadian Rhythms, and Time-Restricted Feeding in Healthy Lifespan. Cell metabolism. 2016;23(6):1048–59. Epub 2016/06/16. 10.1016/j.cmet.2016.06.001 27304506 PMC5388543

[24] (NCD-RisC). NRFC. Worldwide trends in diabetes since 1980: a pooled analysis of 751 population-based studies with 4.4 million participants. Lancet. 2016;387(10027):1513–30. Epub 2016/04/12. 10.1016/S0140-6736(16)00618-8 27061677 PMC5081106

[25] Shetty PS. Nutrition transition in India. Public Health Nutr. 2002;5(1A):175–82. Epub 2002/05/25. 10.1079/PHN2001291 12027282

[26] Gill S, Panda S. A Smartphone App Reveals Erratic Diurnal Eating Patterns in Humans that Can Be Modulated for Health Benefits. Cell metabolism. 2015;22(5):789–98. Epub 2015/09/29. 10.1016/j.cmet.2015.09.005 26411343 PMC4635036

[27] Six BL, Schap TE, Kerr DA, Boushey CJ. Evaluation of the Food and Nutrient Database for Dietary Studies for use with a mobile telephone food record. Journal of food composition and analysis: an official publication of the United Nations University, International Network of Food Data Systems. 2011;24(8):1160–7. Epub 2012/03/06. 10.1016/j.jfca.2011.06.006 PMC3289151 22389554

[28] Six BL, Schap TE, Zhu FM, Mariappan A, Bosch M, Delp EJ, et al Evidence-based development of a mobile telephone food record. Journal of the American Dietetic Association. 2010;110(1):74–9. Epub 2010/01/28. 10.1016/j.jada.2009.10.010 20102830 PMC3042797

[29] Roza AM, Shizgal HM. The Harris Benedict equation reevaluated: resting energy requirements and the body cell mass. The American journal of clinical nutrition. 1984;40(1):168–82. Epub 1984/07/01. 6741850 10.1093/ajcn/40.1.168

[30] Tuomi T, Nagorny CL, Singh P, Bennet H, Yu Q, Alenkvist I, et al Increased Melatonin Signaling Is a Risk Factor for Type 2 Diabetes. Cell metabolism. 2016;23(6):1067–77. Epub 2016/05/18. 10.1016/j.cmet.2016.04.009 27185156

[31] Narayan KM, Boyle JP, Thompson TJ, Gregg EW, Williamson DF. Effect of BMI on lifetime risk for diabetes in the U.S. Diabetes care. 2007;30(6):1562–6. Epub 2007/03/21. 10.2337/dc06-2544 17372155

[32] Huxley R, Mendis S, Zheleznyakov E, Reddy S, Chan J. Body mass index, waist circumference and waist:hip ratio as predictors of cardiovascular risk—a review of the literature. Eur J Clin Nutr. 2010;64(1):16–22. Epub 2009/08/06. 10.1038/ejcn.2009.68 19654593

[33] Rothschild J, Hoddy KK, Jambazian P, Varady KA. Time-restricted feeding and risk of metabolic disease: a review of human and animal studies. Nutr Rev. 2014;72(5):308–18. Epub 2014/04/18. 10.1111/nure.12104 24739093

